# Random egg laying in host nests, rather than egg-matching, explains patterns of cuckoo parasitism: a comment on Zhang *et al*. (2023)

**DOI:** 10.1098/rspb.2023.1018

**Published:** 2023-09-13

**Authors:** Longwu Wang, Wei Liang

**Affiliations:** ^1^ School of Life Sciences, Guizhou Normal University, Guiyang 550001, People's Republic of China; ^2^ Ministry of Education Key Laboratory for Ecology of Tropical Islands, Key Laboratory of Tropical Animal and Plant Ecology of Hainan Province, College of Life Sciences, Hainan Normal University, Haikou 571158, People's Republic of China

The common cuckoo (*Cuculus canorus*) is the most extensively studied brood parasite. It lays eggs in host nests and depends on the host to incubate its eggs and raise its offspring [1]. The high reproductive cost to hosts has resulted in the evolution of means to resist parasitism, including mechanisms for identifying and rejecting cuckoo eggs [2,3]. Theoretically, to reduce the recognition and rejection of cuckoo eggs by hosts, optimality models predict that female cuckoos will choose nests of species with a similar egg colour (hereafter referred to as ‘egg-matching’) as parasitic targets [4] because random laying would be wasteful [1]. Owing to weak evidence for egg-matching and conflicting results, the determinants of host nest selection by cuckoos were largely unresolved [5–7], until Yang *et al*. [8] demonstrated that female cuckoos ignore the colour and morphology of eggs in the host nest during egg-laying. This field study, based on strong video evidence and an effective experimental design, was the first to show that egg-laying in cuckoos is random and not determined by an egg-matching strategy. Subsequently, Yang *et al*. [9,10] provided further evidence for the random egg-laying in common cuckoos and in an additional parasitic system, the plaintive cuckoo (*Cacomantis merulinus*) [11]. Recently, Zhang *et al*. [12] have published an opposing view, suggesting that common cuckoos select nests for parasitism based on an egg-matching strategy (i.e. common cuckoos that lay blue eggs prefer host nests with blue eggs and avoid host nests with pink eggs). However, we suggest that their experimental results do not fully support the egg-matching hypothesis and require further video evidence.

In terms of the natural parasitism rate, Zhang *et al*. [12] found that the common cuckoo selects nests with blue eggs (19.3%, 53/275) more frequently than nests with pink eggs (7.9%, 16/203). This finding was consistent with the egg-matching hypothesis because the common cuckoo lays blue eggs, and thus selecting hosts with blue eggs would reduce the probability of egg identification and rejection by the host due to the highly mimetic egg coloration. The Daurian redstart (*Phoenicurus auroreus*) can recognize and reject non-mimetic eggs [13], and this is expected to substantially increase the success rate of parasitism. However, in the study by Zhang *et al*. [12], an extremely important issue is that the comparison of parasitism rates between blue and pink eggs was only based on nest-boxes with surviving cuckoo eggs, which excluded the majority of blue cuckoo eggs in nest-boxes with pink eggs that were rejected by the hosts. Human observation and avian vision modelling have both demonstrated that the egg colour of the common cuckoo is highly similar to that of host eggs [12]. Thus, cuckoo eggs in nests with blue eggs are largely accepted by the host. Conversely, blue cuckoo eggs laid in a pink host nest would theoretically have an increased chance of being rejected due to the colour difference. The rejection rate of artificial model eggs in pink-egg nests of the host population is approximately 90% ([13], fig. 3). In addition, Yang *et al*. [14] also showed that Daurian redstarts rejected 61% of conspecific eggs with dissimilar morphs (*n* = 23), 100% for white clutches (i.e. white clutches reject 100% of conspecific blue eggs; *n* = 6) and 47% for blue clutches (i.e. blue clutches reject 47% of conspecific white eggs; *n* = 17). Even based on Zhang *et al*. [13] for the same redstart population in their study, only approximately 10% of cuckoo eggs survive in pink nests. Comparing surviving parasitic eggs in blue nests, which receive half of the total eggs (or less), would undoubtedly lead to the interpretation that blue nests are preferred by the common cuckoo. This is an example of the classic ‘survivorship bias’ [15], in which only a subset of results, based on a selection process, are included in analyses. Because cuckoo eggs that were rejected in the pink host nests were excluded from analyses in this study, they essentially became ‘silent’ data. The probability of real cuckoo eggs being accepted by hosts with blue eggs should be higher than that for hosts with pink eggs. It is unclear whether survivorship bias is an issue in the study by Zhang *et al*. [12] because we cannot rule out the possibility that the authors might have overlooked instances where redstarts quickly rejected mismatched cuckoo eggs. Therefore, without knowledge of how quickly redstarts typically reject eggs, survivorship bias is an issue that could potentially influence conclusions drawn from the observational data; however, this key information was not provided in the studies by Zhang *et al*. [12,13]. In addition, in this population, substantially more individuals laid blue eggs than pink eggs (56.1% versus 43.9%, *n* = 513) [13], which may also contribute to the higher percentage of parasitism in nests with blue eggs than pink eggs.

In terms of field experiments, the authors established a triplet design by presenting a dummy clutch of each colour morph adjacent to active redstart nests. They concluded that cuckoos prefer to lay eggs in nests with blue eggs; however, this involves two key issues. (i) As mentioned above, if this analysis was affected by ‘survivorship bias’, some cuckoo eggs might have been possibly rejected before they were detected by the researchers. Although the authors checked the nest-boxes daily [12], the interval between daily checks may have been sufficient for the host to reject cuckoo eggs. Cuckoos usually lay eggs in the afternoon or at dusk [16,17]. The authors started checking nests one by one after 17.00 and in the next morning at 8.00–9.00, suggesting that some parasitized cases could have been missed. For example, we assume that nests checked at 17.00 might have been parasitized after/between 17.00–20.00 but were not rechecked until the following day, suggesting that cuckoo eggs could have been rejected by the host, especially in pink nests. The time gap was at least 4–7 h (sunrise was approx. at 3.50 and sunset was at 20.00 in the breeding season in this study site of northeast China) between the checking action of the researchers, and this time gap increased the chance and uncertainty for cuckoo egg rejection by hosts. Thus, the lack of 24/7 surveillance was an important limitation of the study. (ii) There is a lack of direct evidence to confirm that the female cuckoo switches to a blue nest to lay eggs when encountering a nest of pink eggs. This would confirm that the cuckoo does discern the colour of eggs in the nest and targets blue nests for parasitism. Therefore, the lack of video footage documenting the nest selection process is another key limitation of the study.

In fact, studies on multiple species support the hypothesis of random egg-laying by parasites, rather than egg-matching based on egg colour. Antonov *et al*. [18] used avian vision modelling and image analyses to compare cuckoo eggs in naturally parasitized nests of the marsh warbler *Acrocephalus palustris* and their nearest unparasitized conspecific neighbours, obtaining no evidence that cuckoos parasitize nests where their eggs better match the host eggs. Yang *et al*. [19], in a study of parrotbill host (*Paradoxornis alphonsianus*) populations, similarly found no evidence of the hypothesis that cuckoos lay eggs based on egg colour matching. Evidence for random egg-laying has been obtained in studies of the common cuckoo and Oriental reed warbler (*Acrocephalus orientalis*), including clear video evidence that egg-laying processes in the common cuckoo occur in very short periods (3–10 s) and follow random patterns, primarily targeting host identity and active nests for parasitism [8–11].

Experimental support for random egg-laying is increasing in different host–cuckoo parasitism systems. First, in field experimental parasitism, female cuckoos laid a large number of eggs in nests with black or white model eggs, with no matching based on egg colour [16,20]. Second, common cuckoos lay abundant eggs in nests with model nestlings, and the nestling model is even less compatible with cuckoo eggs [21]. Cuckoos sometimes parasitize host nests with real nestlings [22]. Even if such cases are relatively rare, it is important to note that cuckoos sometimes make mistakes. Third, cuckoos often lay eggs in empty nests. The absence of any object in the nest, with no reference for matching, indicates that cuckoo egg matching is not an important strategy [16,17]. In particular, female cuckoos lay a large number of eggs on the outer edge of common redstart (*Phoenicurus phoenicurus*) nests, causing many cuckoo eggs to hatch unsuccessfully [23]. The cuckoo approaches the host nest with its tail and quickly completes the egg-laying process at the nest entrance, probably because of the small nest entrance or the likelihood of a fierce attack by the host [23,24]. This cavity-nesting behaviour suggests that the cuckoo does not check the egg pattern in time during parasitism. Fourth, observations of multiple parasitisms indicate that female cuckoos that arrive late could not have used an egg-matching strategy. In the field, researchers found two or more cuckoo eggs in the same nest [16,17,25], and none of these eggs were laid by the same cuckoo individual. Moreover, quintuple parasitism of a cuckoo host has been reported [26]. Quantitatively, the presence of host eggs and eggs of different cuckoo individuals within the same nest suggests that egg matching does not occur. Finally, the theoretical analysis revealed that cuckoos should lay eggs randomly as almost all of their parental investment is devoted to producing eggs, suggesting that they may lay eggs indiscriminately. If cuckoos chose egg-matching with their own egg appearance, they would cause constraints on the flexibility of their progeny to exploit and adapt to a new host. Furthermore, egg matching would increase the risk of detection of the female cuckoo by hosts, which might upregulate anti-parasitic defences [19].

In summary, both theoretical [19] and experimental studies [8–11] indicate that the common cuckoo probably uses a random egg-laying strategy. Conversely, the egg-matching strategy is likely to be maladaptive and is not sufficiently supported by Zhang *et al*. [12]. However, we also emphasize that their study is valuable as it provides important insights into enhancing our understanding of the interactions between cuckoos and their hosts regarding nest selection.

## Data Availability

This article has no additional data.
